# Estimating nearshore coral reef-associated fisheries production from the main Hawaiian Islands

**DOI:** 10.1371/journal.pone.0195840

**Published:** 2018-04-16

**Authors:** Kaylyn S. McCoy, Ivor D. Williams, Alan M. Friedlander, Hongguang Ma, Lida Teneva, John N. Kittinger

**Affiliations:** 1 Joint Institute of Marine and Atmospheric Research, University of Hawaiʻi at Mānoa, Honolulu, Hawaiʻi, United States of America; 2 Fisheries Ecology Research Lab, University of Hawaiʻi at Mānoa, Honolulu, Hawaiʻi, United States of America; 3 Ecosystem Sciences Division, Coral Reef Ecosystem Program, Pacific Islands Fisheries Science Center, Honolulu, Hawaiʻi, United States of America; 4 Pristine Seas, National Geographic Society, Washington, D.C., United States of America; 5 Fisheries Research and Monitoring Division, Insular Fisheries Monitoring Program, Pacific Islands Fisheries Science Center, Honolulu, Hawaiʻi, United States of America; 6 Conservation International, Center for Oceans, Honolulu, Hawaiʻi, United States of America; 7 Arizona State University, Center for Biodiversity Outcomes, Julie Ann Wrigley Global Institute of Sustainability, Life Sciences Center, Tempe, Arizona, United States of America; 8 Conservation International, Betty and Gordon Moore Center for Science, Arlington, Virginia, United States of America; Leibniz Centre for Tropical Marine Research, GERMANY

## Abstract

Currently, information on nearshore reef-associated fisheries is frequently disparate or incomplete, creating a challenge for effective management. This study utilized an existing non-commercial fishery dataset from Hawaiʻi, covering the period 2004–13, to estimate a variety of fundamental fishery parameters, including participation, effort, gear use, and catch per unit effort. We then used those data to reconstruct total catches per island. Non-commercial fisheries in this case comprise recreational, subsistence, and cultural harvest, which may be exchanged, but are not sold. By combining those data with reported commercial catch data, we estimated annual catch of nearshore reef-associated fisheries in the main Hawaiian Islands over the study period to be 1,167,758 ± 43,059 kg year^-1^ (mean ± standard error). Average annual commercial reef fish catch over the same time period—184,911 kg year^-1^—was 16% of the total catch, but that proportion varied greatly among islands, ranging from 23% on Oʻahu to 5% on Molokaʻi. These results emphasize the importance of reef fishing in Hawaiʻi for reasons beyond commerce, such as food security and cultural practice, and highlight the large differences in fishing practices across the Hawaiian Islands.

## Introduction

In the Hawaiian islands, the diverse nearshore coral reef-associated fisheries support a range of commercial, recreational, and subsistence activities that employ multiple gear types, and harvest a wide variety of reef and estuarine finfishes, invertebrates, and schooling coastal pelagic species [1,2]. Communities in Hawai‘i rely substantially on these fisheries for economic, social, and cultural services, including important livelihood and food provisioning functions [1,3]. Total annual value of Hawaiʻi’s nearshore coral reef-associated fisheries is estimated to be between $10.3 and $16.4 million, with the majority of value associated with non-commercial fishing ($7.2 - $12.9 million), which amounts to more than 7 million meals annually [4].

Maintaining the diverse benefits from fisheries depends on implementing sustainable management practices. To achieve sound management, changes in fish catch and fish populations must be measured with high accuracy to determine the ecosystem health in association with fishing pressure. Nearshore coral reef-associated fisheries are particularly challenging to assess and to manage for several reasons: small-scale, non-commercial fisheries often use numerous gear types over a wide geographic range by many fishers, making effort and catch difficult to accurately assess. Another complication is that much of that catch remains un- or under-reported in existing surveys and mandatory reporting systems, if it is available at all [5–8].

Nearshore catch in the main Hawaiian Islands has declined substantially over the past 100 years [8], and several target stocks are depleted below benchmark sustainability levels [9,10]. Target fish biomass is low in comparison to baseline estimates (before human impact) for the main Hawaiian Islands [10,11]. Given these levels of depletion, it is more imperative that the nearshore fisheries are sustainably managed. This includes using any available data sources to better understand these complex fisheries.

Currently, there are two main data sources available at the statewide level, including commercial catch data, which is self-reported by commercial fishers, and non-commercial data, which uses information from telephone and intercept surveys. These datasets are typically summarized and reported on at the statewide scale. However, a number of recent studies in Hawaiʻi have highlighted large differences in apparent fishing pressure and stock status among different parts of the main Hawaiian Islands [10,12], indicating the importance of properly quantifying fishing pressure at smaller spatial scales. Further, despite significant investment in data collection for nearshore fisheries, there are no comprehensive estimates for production from nearshore fisheries in Hawaii.

The purpose of this study is to develop detailed estimates of production and other key fishery parameters for nearshore fisheries in Hawai‘i. Our goals are to: (i) Develop basic information about key fishery parameters such as rates of participation, effort, gear preference, and catch per unit effort (CPUE) per island, using the detailed non-commercial fishery information; (ii) Estimate non-commercial reef fish catches at the island-scale; and (iii) Combine and compare commercial and non-commercial fishery catch at island and statewide scales, developing a first of its kind total production estimate from these fisheries. Collectively, this will greatly improve the utility of the available nearshore fishery data, and help to fill critical gaps in fisheries knowledge necessary to better manage Hawaiʻi’s nearshore coral reef-associated fisheries.

## Methods

### Study area

The main Hawaiian Islands are comprised of the eight larger islands of Niʻihau, Kauaʻi, Oʻahu, Molokaʻi, Maui, Lānaʻi, Kahoʻolawe, and Hawaiʻi, and 124 small islands, reefs, and shoals [13]. Located in the middle of the Pacific Ocean and spanning 644 km, it is one of the most remote populated areas in the world [13], with the closest island group 1,800 km away (http://islands.unep.ch). The islands included in these analyses are Kauaʻi, Oʻahu, Molokaʻi, Maui, Lānaʻi, and Hawaiʻi, as there were no non-commercial data for the islands of Niʻihau and Kahoʻolawe. Waters around Kahoʻolawe are a no-take marine reserve (except for limited take for cultural purposes and on-island consumption), and non-commercial reef fishing data are not available from the lightly populated island of Niʻihau, as access to the island is restricted for non-residents, meaning intercept surveys were not conducted.

### Taxa of interest

Our primary interest was to understand the nearshore reef-associated fishery; therefore, we restricted our catch estimates to nearshore reef-associated finfish species only. We excluded pelagic species such as tuna (Thunnini), billfish (Istiophoridae), and wahoo (*Acanthocybium solandri*), and bottomfishes (e.g. *Etelis* spp., *Pristipomoides* spp.). We also excluded schooling coastal pelagic species—primarily mackerels (*Decapterus* spp.) and bigeye scad (*Selar crumenophthalmus*)—which are harvested seasonally in nearshore waters, but are primarily pelagic species and have highly variable catch in space and time. The complete list of species included in this analysis is given in Supporting Information, S1 Table.

### Data sources

Data for these analyses consisted of non-commercial and commercial fishing data and came from two data sources. Non-commercial fishing data were derived from the Marine Recreational Information Program (MRIP) surveys [14], hereafter referred to as MRIP data. Western Pacific Fisheries Information Network provided commercial fishing data, referred to as commercial marine license (CML) data (https://www.pifsc.noaa.gov/wpacfin/). No names or other personal information were included in the data available to us—all information provided was anonymous. Both datasets are currently available in summary form at the state level, by year. This study synthesized the raw data at a smaller, island scale.

#### Commercial marine landings data–CML

In Hawaiʻi, any fisher who sells any part of their catch is required to have a commercial marine license, which through the course of our study, was readily available for payment of a $50 annual fee. The State of Hawaiʻi requires all license holders to report their catch and effort monthly, regardless of whether the catch is sold or not. License holders are further required to submit a zero report if nothing was caught or even if they did not fish in a particular month. Thus, all CML data is self-reported, with mostly little or no verification. However, a Civil Resource Violation System (CRVS) has been in place since 2009 (http://dlnr.hawaii.gov/apo/), and CML holders can be penalized for delinquencies in reporting including, potentially, losing their license (http://dlnr.hawaii.gov/dar/fishing/commercial-fishing/). The commercial data used here were obtained from the Western Pacific Fisheries Information Network (https://www.pifsc.noaa.gov/wpacfin/) as total reported catches by family for all reef-associated fishes between 2004 and 2013.

The summation in this study differs from the publicly available data, which summarize reef fish or inshore total catch (or species group) by year statewide, or by county, or by fish aggregating device (FAD). This study compiles total catch at the island scale. This assessment focused on nearshore reef-associated fishes; therefore, effort and CPUE data from fishing methods that targeted offshore species, such as deep-water handline, tuna handline, vertical line, and aku (skipjack tuna–*Katsuwonus pelamis*) boat methods were excluded from the analyses. Because they are supposed to be a complete record of commercial fish catch (i.e. not a sub-sample), there is no statistical error associated with the CML data.

#### Non-commercial data–MRIP data overview

Non-commercial fishing data from the main Hawaiian Islands are gathered by the National Oceanic and Atmospheric Administration (NOAA) and Hawaiʻi’s Division of Aquatic Resources (HDAR)’s Hawaiʻi Marine Recreational Fishing Survey (HMRFS). This is part of a national program, the Marine Recreational Information Program (MRIP), previously known as Marine Recreational Fisheries Statistics Survey (MRFSS). Summaries of the MRIP catch and effort data have been available at a state level [14] and from the MRIP website (http://www.st.nmfs.noaa.gov/st1/recreational/MRIP_Survey_Data/)], but were not previously available at a smaller scales. As described above, a core goal of this study is to use the raw MRIP survey data (available at http://www.st.nmfs.noaa.gov/st1/recreational/MRIP_Survey_Data/) to reconstruct catch and effort estimates at island-scale.

The MRIP survey consists of two components: a telephone survey to estimate fishing effort by household, and an intercept survey of fishers at access sites to estimate catch and CPUE. Total catch expansions are developed using catch rate estimates from the intercept surveys and effort estimates derived from the phone surveys. The HMRFS program began on Oʻahu in 2001, and was extended to include neighbor islands between 2002 and 2004. In 2006, the program evolved into the MRIP program. Here we use data from 2004–2013 to make comparisons among all islands covered by MRIP: Kauaʻi, Oʻahu, Molokaʻi, Lānaʻi, Maui, and Hawaiʻi [15].

Bimonthly landline telephone surveys are conducted using a random-digit dialing system. Households are asked if anyone fished in the past 2 months, and if so, how many times. Details from each trip are recorded, including mode or platform (boat or shore) and gear type(s) used. The resulting information yields the number of trips per gear type per fisher for that 2-month period, called a wave [15]. In total, we had data from 117,327 telephone interviews, 12,042 of which were from fishing households who provided information on a total of 58,687 fishing trips. One of the questions fishers are asked is if they are commercial fishers. The MRIP data we use is only from fishers who answered no to that question.

Between 2004 and 2013, a total of 23,798 Intercept surveys were conducted across 25 sites on Kauaʻi, 55 sites on Oʻahu, 11 on Molokaʻi, 16 on Maui, and 41 on Hawaiʻi Island. Intercept sites were chosen based on fishing pressure at the site, estimated by number of fishers present during a time interval at the site. For each survey, interviewers approached fishers as they were leaving the shore or boat ramp and requested an interview about their fishing effort (primary gear used, durations of fishing) and to quantify their catch (number, species, and sizes). Surveys were conducted during the daytime and therefore miss some nighttime fishing activity. Some of the nighttime fishing activity is captured by the interviews that intercept fishers returning from night fishing trips the following morning. There are no intercept sites on the island of Lānaʻi, but we averaged catch data from the other islands to calculate substitute CPUE for that location.

For this study, we extract and synthesize four types of information from the MRIP data at island-scale;—number of trips, trip types (distribution of trips among platforms—boat or shore—and gears—line, net, or spear), gear hours (duration of fishing trip per gear), and CPUE (per platform and gear). Our methods demonstrating how we obtain and use those data to reconstruct island-scale catches and propagate the uncertainty in those estimates are described in more detail below.

#### MRIP data–number of fishing trips and trip types

We calculated effort in terms of number of fishing trips and types of those trips (i.e., which gears were deployed) from the telephone survey data. Specifically, using all completed telephone interviews per island and wave, we calculated participation rate, *pr*, as the proportion of households in which someone fished in the wave (i.e., 2-month period), and *tr*, the mean number of trips per fishing households. Total number of fishing trips, *FT*, was calculated using the formula:

$${FT}_{iw}={pr}_{iw}*{tr}_{iw}*{hh}_{i}$$ Eq 1

in which *hh*_*i*_ is the number of households per island taken from the US Census (http://www.census.gov/2010census/), and *i* and *w* represent different islands and waves (with there being 60 2-month waves in the 10-year dataset).

If a participant reported fishing activity in the previous wave during a telephone interview, they were then asked for more information, including the platform (boat or shore) and gear used. We classified gears into 4 groups; ‘line’, ‘net’, ‘spear,’ or ‘not-reef. ‘Not-reef’ refers to all trips and gears that rarely or never target reef fishes (details of how we classified reported reef fishing gears into 4 broad reef fishing categories are given in supporting information S2 Table). If, as was often the case, more than one gear type was listed per trip, each gear was weighted equally, i.e., if three gears were reported for a trip, then each gear would be considered to have been used for 1/3 of that trip. For each combination of island, gear, and year, we then calculated the proportion of trip in each type (i.e. trip type, *TT*) as:

$${TT}_{iyg}=\frac{{FT}_{iyg}}{{FT}_{iy}}$$ Eq 2

in which *FT* is the total number of fishing trips (including partial trips), and *i*, *y*, and *g* are island, year, and gear, respectively. The sum of *TT* for each island and year, therefore, equals 1, and the product of *TT* and *FT* is the number of trips per gear type.

#### MRIP data–CPUE and gear hours (duration of fishing trips)

We used data from the intercept surveys to calculate reef fish CPUE and trip duration (i.e., hours fished per trip per gear). We chose to pool those data over the entire 10-year time frame due to small sample sizes for several combinations of island, platform, and gear at smaller time frames–particularly for boat fishing (S3 Table). As our goal was to calculate CPUE in terms of catch weight, we used the approach described in [14] to generate weight estimates for cases in the intercept data where catch composition and number but not weights were recorded–that is, we used average substitute weights for species relative to platform and gear type. We then used those data to calculate the total weight of reef fishes (taxa listed in S1 Table) per fishing trip.

For gear hours, *GH*, we simply calculated the mean and standard error (SE) of trip duration for each combination of island, platform, and gear. Because CPUE is a ratio, we used the approach described in [16] to calculate mean and variance, namely:

$${CPUE}_{ig}=\frac{{\bar{c}}_{ig}}{{\bar{e}}_{ig}}$$ Eq 3

where $\bar{c}$ is mean catch (kg), $\bar{e}$ is mean effort (hr), and *i* and *g* are island and gear type, respectively. Variance of CPUE mean was calculated as:

$$V({CPUE}_{ig})={CPUE}_{ig}^{2}*\frac{1}{n_{ig}}(\frac{var(c_{ig})}{{\bar{c}}_{ig}^{2}}+\frac{var(e_{ig})}{{\bar{e}}_{ig}^{2}}-\frac{2\;cov(c_{ig},e_{ig})}{{\bar{c}}_{ig}*{\bar{e}}_{ig}})$$ Eq 4

where n is the number of catch interviews.

#### MRIP data–total catch estimates

To generate total catch, *TC*, we combined the information on number of trips, gear type, CPUE, and gear hours using the formula:

$${TC}_{igw}={FT}_{iw}*{TT}_{iyg}*{GH}_{ig}*{CPUE}_{ig}$$ Eq 5

**Fig 1:**
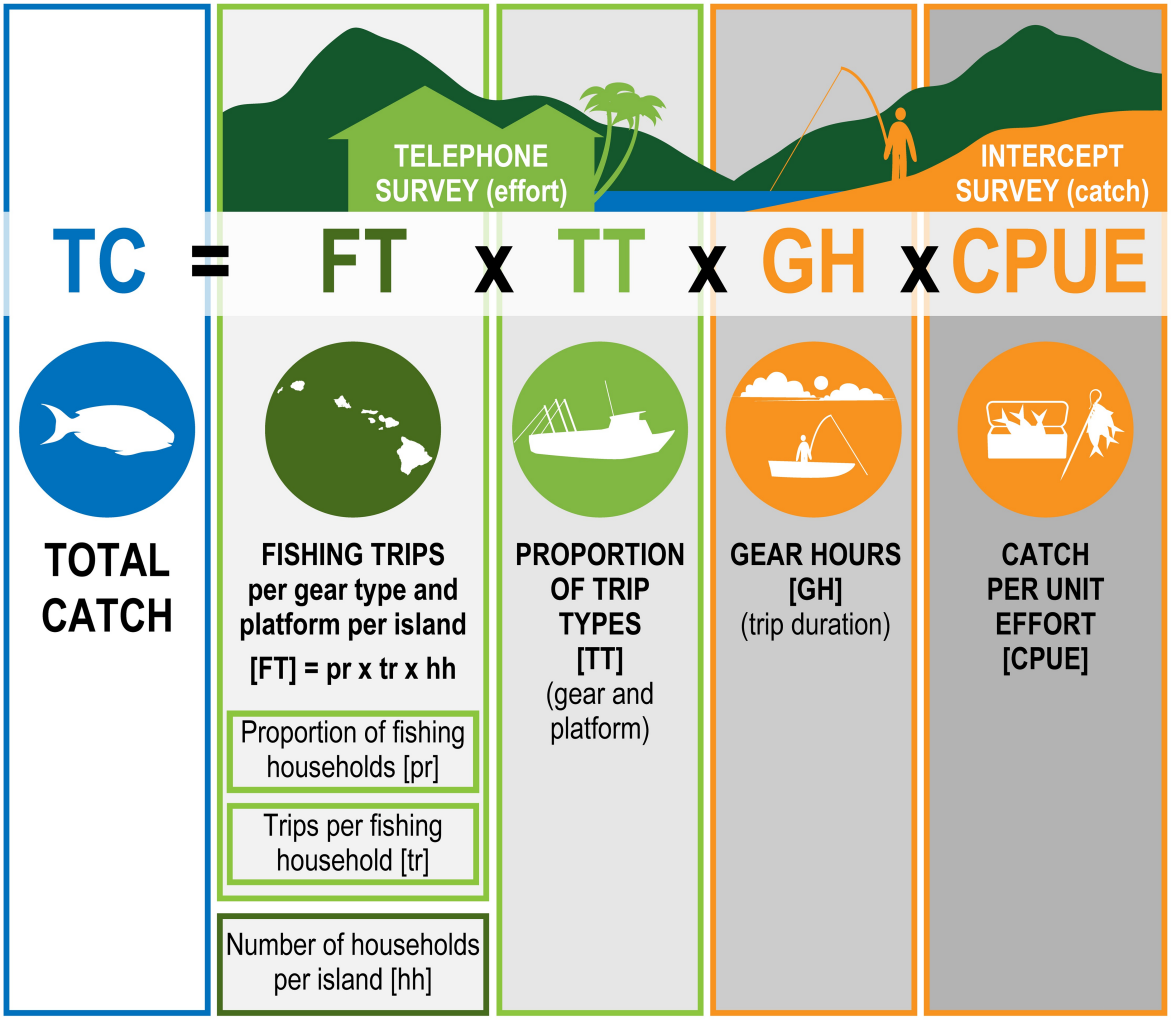
Total catch calculations from MRIP surveys. Information came from telephone effort surveys (outlined in green) or intercept surveys (outlined in orange), and correspond with the variables in Eq 5.

We then summed total catch for all reef gears (line, net, and spear) for all combinations of island and year (Fig 1). An estimate of uncertainty of total catch estimates was generated using a Monte Carlo approach that propagated the uncertainty associated with each of number of trips per wave (FT), trip-duration (GH), and CPUE. Specifically, we performed 5,000 iterations of a loop that calculated total catch weight for each combination of gear, island, year, wave, and platform. In each iteration, total number of trips for island and wave combination (FT) was derived by generating a pseudo-household telephone survey of the same size as the actual telephone survey, with number of fishing households randomly pulled from a binomial distribution (p = probability of an interviewed household being a fishing household, i.e., the proportion of households that reported fishing activity for that island and wave in the original data). The number of trips per each of those households was generated by resampling with replacement from the interviews with fishing households in that island and wave combination. Trip types were then allocated the TT values for that island and year. Trip duration (GH) and CPUE for each gear, island, and platform combination were pulled from normal distributions using the mean and standard error of those generated as described above. Total catch was then calculated by multiplying number of trips, trip duration, and CPUE to generate total simulated catch for each island, wave, gear, and platform combination (Eq 5), and those were aggregated into higher levels, such as average annual catch for each island. We then used mean and variance of catch estimates from the Monte Carlo simulation to quantify estimated overall catch and uncertainty.

### Total catch calculations (commercial + non-commercial)

Total catch was calculated by combining commercial and non-commercial catch from two data sources. The commercial catch has no associated standard error as it is considered a complete tally of the fishery; therefore, standard error for the combined total catch estimates was the same as for the MRIP data only.

### Estimating reef fishery yield

In order to generate an estimate of yield, or fish catch per unit of reef, we divided estimated total annual reef fish catch by a proxy for the amount of reef habitat, hard-bottom habitat in < 30m, taken from an earlier study [11].

## Results

### Fishing effort

The mean percent of households with anyone who fished in the past two months (wave) ranged from 6% on Oʻahu to 24% on Molokaʻi (Table 1). The mean number of fishing trips per fishing household ranged from ~9 (Oʻahu) to ~15 (Molokaʻi, Table 1). By combining those data with information on the number of households, we estimated fishing trips per island per 2-month wave to range from 2,713 on Lānaʻi to 157,332 on Oʻahu (Table 1). In aggregate, this equates to over 2 million fishing trips per year.

**Table 1:** Recreational fishing effort. Data come from MRIP telephone surveys in 2004–2013. Data are summarized as mean and standard deviation of values per wave (i.e., for the 60 2-month periods in that 10-year period). Participation rate is proportion of households in which someone fished in the preceding wave; trips per household represents the number of fishing trips per fishing household in that period. Total # trips was calculated using Eq 1. Number of households ranged from 63,209 to 64,909 for Hawaiʻi Island, 21,968 to 22,390 for Kauaʻi, 1,068 to 1,074 for Lānaʻi, 43,505 to 49,080 for Maui, 2,525 to 2,561 for Molokaʻi, and 303,794 to 309,803 for Oʻahu.

	Participation and effort per wave (2-month period)		
Island	Participation rate (% households that fished)	Trips per fishing household	Total # trips
Hawaiʻi	12.8 (2.3)	10.5 (2.7)	85,382 (25,382)
Kauaʻi	12.8 (2.6)	14.1 (6.8)	39,868 (19,280)
Lānaʻi	19.5 (10.8)	11.4 (10.7)	2,713 (2,627)
Maui	9.4 (2.1)	10.5 (3.7)	45,447 (15,843)
Molokaʻi	24.1 (7.1)	14.7 (8.7)	9,230 (6,687)
Oʻahu	5.6 (1.4)	9.1 (3.1)	157,332 (73,612)

For all islands, the majority of fishing trips targeted reef fishes from shore (65.9% to 83.2% of all trips, Table 2). The single most common fishing trip type at all islands was line fishing from shore, which comprised between 44.7 and 67.4% of all fishing trips (Molokaʻi and Kauaʻi respectively, Table 2). The second most common fishing trip type was boat fishing not targeting reef fishes, which constituted 14.0% to 21.9% of trips (Table 2). Shoreline spearing and netting were the next most common trip types, each making up between ~3 and 12% of all trips at any particular island (Table 2).

**Table 2:** Recreational trip types. Data come from MRIP telephone surveys in 2004–2013. Data are summarized as mean and standard deviation per island and wave of the percentages of fishing trips per combination of platform (boat or shore) and gear as defined by Eq 2. Gears have been pooled into line, net, spear, or not-reef (offshore, pelagic or bottomfish fishing), as described in S2 Table.

		Proportion of Fishing Trips (%)					
Platform	Gear	Hawaiʻi	Kauaʻi	Lānaʻi	Maui	Molokaʻi	Oʻahu
Boat	Line	3.1 (0.6)	3.2 (1.9)	2.2 (2.0)	4.0 (4.1)	3.6 (3.1)	4.9 (2.7)
	Net	0.2 (0.2)	0.3 (0.5)	0.0 (0.0)	0.1 (0.1)	3.6 (3.8)	0.3 (0.8)
	Spear	1.2 (0.6)	1.3 (1.6)	0.3 (0.8)	1.2 (0.5)	4.6 (2.5)	1.4 (0.8)
	Not-reef	17.4 (2.5)	14.3 (4.9)	14.0 (6.2)	15.8 (4.2)	21.9 (5.0)	15.6 (3.3)
Shore	Line	65.6 (3.2)	67.4 (6.5)	65.9 (10.6)	61.3 (5.7)	44.7 (10.4)	66.8 (5.0)
	Net	3.3 (1.0)	7.1 (7.8)	7.1 (7.4)	5.2 (3.0)	11.1 (8.8)	2.7 (1.3)
	Spear	9.0 (2.5)	6.3 (2.6)	10.2 (3.4)	12.1 (1.7)	10.0 (4.3)	8.3 (2.0)
	Not-reef	0.2 (0.3)	0.1 (0.2)	0.3 (1.0)	0.2 (0.3)	0.4 (0.9)	0.0 (0.0)

Combining trip types and number of trips gives a total of ~2.0 million fishing trips per year in the Main Hawaiian Islands. ~1.7 million of those trips were classified by us as reef fishing trips, nearly half of which were on Oʻahu.

### Catch per unit effort (CPUE)

CPUE tended to be highest for net fishing, followed by spear, and then line. For line fishing, CPUE was generally higher for boat fishing than shore fishing (Table 3). The highest CPUE value for any combination of platform, gear, and island was 1.73 kg hr^-1^ for boat spear fishing around Maui, and the lowest value was 0.06 kg hr^-1^ for line fishing from shore at Oʻahu (Table 3).

**Table 3:** Recreational CPUE per island, platform, and gear. Data shown are mean and standard error (SE) of CPUE by island for each combination of island, platform, and gear in intercept surveys by MRIP between 2004–13. Eqs 3 and 4 were used to calculate mean and variance of CPUE values. Reef fishes are taxa as defined in S1 Table. Note that as there were no intercept surveys on Lānaʻi, CPUE data for there are assumed to be the average from other islands. Lānaʻi CPUE SE was generated using the average precision (SE/mean) from all other islands. The number of catch interviews per combination of island, platform, and gear is given in S3 Table.

		CPUE (kg Reef Fish / hr fished)					
Platform	Gear	Hawaiʻi	Kauaʻi	Lānaʻi	Maui	Molokaʻi	Oʻahu
Boat	Line	0.12 (0.02)	0.07 (0.02)	0.26 (0.06)	0.07 (0.02)	0.73 (0.26)	0.30 (0.04)
	Net	0.66 (0.43)	1.25 (0.89)	0.85 (0.58)	0.00 (0.00)	0.80 (0.34)	1.53 (1.00)
	Spear	0.90 (0.21)	0.43 (0.16)	0.85 (0.20)	1.73 (0.39)	0.83 (0.13)	0.38 (0.07)
Shore	Line	0.15 (0.02)	0.08 (0.01)	0.13 (0.01)	0.10 (0.01)	0.25 (0.03)	0.06 (0.00)
	Net	0.42 (0.06)	0.38 (0.17)	0.82 (0.27)	0.84 (0.24)	1.56 (0.57)	0.90 (0.38)
	Spear	0.67 (0.08)	0.39 (0.17)	0.42 (0.09)	0.38 (0.06)	0.32 (0.06)	0.34 (0.05)

The lack of intercept data from Lānaʻi required us to use averaged data from other islands. However, as Lānaʻi fishing trips made up less than 1% of total statewide trips (Table 1), any associated error would have had little overall impact on statewide catch estimates.

### Total catch estimates

We estimated mean total nearshore reef-associated catch to be 1,167,758 ± 43,059 kg year^-1^ for the main Hawaiian Islands. The non-commercial catch from the MRIP estimates were combined with the CML catch estimates to generate a yearly average from 2004–2013 (Table 4). At the island level, mean annual catch ranged from 10,437 ± 974 kg on Lānaʻi to 462,255 ± 27,341 kg on Oʻahu.

**Table 4:** Total catch by island in kg year^-1^. Values show estimates of commercial and non-commercial catch for each island, as well as a statewide total and combined catch. Yearly estimates are from the time frame of 2004–2013. ‘Other’ for CML comes largely from Niʻihau, which has a resident population of ~100 people and is ~30km from Kauaʻi; but also includes catch from offshore banks and pinnacles.

	Mean and SE of Annual Catch (kg)			
Island	Non-commercial (MRIP)	Commercial (CML)	Combined	MRIP:CML
Hawaiʻi	321,131 (28,164)	30,612	351,743	10.5
Kauaʻi	78,374 (9,644)	10,638	89,012	7.4
Lānaʻi	9,063 (974)	1,374	10,437	6.6
Maui	142,846 (10,893)	24,289	167,135	5.9
Molokaʻi	77,654 (10,082)	4,251	81,905	18.3
Oʻahu	353,780 (27,341)	108,445	462,225	3.3
Other		5,301		
State-wide	982,847 (43,059)	184,911	1,167,758	5.3

Non-commercial total catch was substantially higher than the commercial catch of nearshore reef fish for each island, making up approximately 84% of the total catch. Relative to commercial catch, recreational catch per island varied from 3.3 times as much on Oʻahu to 18.3 times as much for Molokaʻi (Table 4).

### Reef fishery yield

Reef fish yield across the main Hawaiian Islands, calculated as total estimated reef fish catch divided by area of hard-bottom habitat in < 30m of water, was 1.19 metric tons km^-2^ year^-1^, with values ranging from 0.35 for Lānaʻi to 2.09 for Hawaiʻi Island. The value was 0.49 for Kauaʻi, 1.50 for Maui, 0.64 for Molokaʻi, and 1.84 metric tons km^-2^ year^-1^ for Oʻahu.

## Discussion

By combining different data sources, we have been able to generate the most complete summary of nearshore coral reef-associated fisheries in Hawaiʻi to date. Our results highlight the dominance of non-commercial fisheries, the magnitude of combined nearshore fisheries, and the disparity of fishing practices between islands. These results are considered in more detail below.

First, our estimates of reef fishery production highlight the importance of non-commercial fisheries as the dominant production mode in these environments. Non-commercial fisheries produce more than 5 times the catch from commercial fisheries, and account for approximately 84% of the total production (Table 4). Clearly, non-commercial fisheries are important in Hawaiʻi for both culture and food security [17–19]. The difficulty in properly monitoring and assessing non-commercial critically important reef fisheries for food provisioning poses a challenge for management [20–22]. The relatively low contribution of commercial fishing to total reef fish catch in Hawaiʻi that is evident from this research and other studies [23,24], emphasizes the need for management agencies to account for these harvesting activities as a priority for management and data reporting. In addition, it would be extremely valuable to routinely generate and report fishery data at island- or smaller spatial scales. Reef fish management in Hawaiʻi already varies among islands, as evidenced by the recent adoption of Maui-specific bag and size limits for parrotfishes and goatfishes (http://dlnr.hawaii.gov/dar/fishing/fishing-regulations/, Hawaiʻi Administrative Rules, Title 13.4, Ch. 95.1).

Second, our research reveals there is considerable diversity in fishing intensity, and gear usage among islands. This is likely due to the considerable variability among the Hawaiian Islands in a range of complex social, ecological and biophysical factors. For example, at several islands there are large areas of reef habitat that are relatively inaccessible to shore and boat fishers through large parts of the year [12]. There are also a large variety of reef types and nearshore profiles; Oʻahu has relatively large areas of shallow reef flats that are conducive to shore net fishing, whereas Hawaiʻi and other islands tend to have much steeper and narrow reef shelfs. There are also large differences in participation rate (e.g. on average 5.6% of Oʻahu households engaged in fishing per 2-month period compared to 24.1% on Molokaʻi), which affects total catches, yields, and relative proportions of commercial and non-commercial reef fisheries among islands. The variability in catch and gear usage we report is consistent with recent studies that have demonstrated the high variability in coral reef fisheries among different parts of the Hawaiian Islands [4,25]. This diversity among locations points to the potential utility of place-based management approaches, which have been implemented throughout the Pacific [26–28], and with some indications of success in Hawaiʻi [3,29]. Within Hawaiʻi, there is growing interest and support for community co-management based on traditional ecological knowledge and strong participation of communities surrounding the fisheries [30,31]. These place-based management approaches would benefit from tailored fisheries monitoring approaches or creel programs to inform catch and effort controls developed at the community level [32]. In addition to community level management, there is also a need for regional (i.e. island) management structures in order to ensure sustainability, due to reef fish mobility and distribution of resources [33].

Based on our results, yields from reef fisheries in Hawaiʻi are low to moderate compared to values reported from elsewhere in the region. Our estimates of yield of coral reef fishes in the main Hawaiian Islands varied from ~0.5 metric tons km^-2^ around Lanaʻi, Molokaʻi, and Kauaʻi to a little over 2 metric tons km^-2^ around Hawaiʻi Island, which, compared to other main Hawaiian Islands, is moderately populated but is steep-sided and thus has a narrow band of shallow reef habitat around it. Those yield values are similar to recent estimates for Guam [34], but are on the low to moderate end of reef fishery yields reported from other locations, which vary from ~0.1 to > 30 T km^-2^, with an average of ~3 T km^-2^ [35].

Production and CPUE estimates for reef fisheries are rare, and our analysis demonstrates that investments in data collection and analysis can be used to better assess the value and performance of reef fisheries. The largest part of our study involved using basic data on fishing effort and catch from the MRIP program to estimate catches via different combinations of gear, platform, island, and time period, and use those to characterize fisheries at island-scale. The fact that our reconstructed statewide annual non-commercial reef fish catch of 982,847 ± 43,059 kg year^-1^ was within 3% of the previously reported MRIP statewide catch expansion of 1,014,380 kg year^-1^ for the same period [14] suggests that our process did not introduce substantial bias in the reconstructed catch estimates.

There are a number of known limitations in the MRIP data [14,36–38], including the inherent difficulty in quantifying the highly diverse, often small-scale and spatially-dispersed non-commercial reef fishery in Hawaiʻi. Over the last few decades, there have been a number of creel surveys focused around different communities [25]. Properly implementing creel surveys requires considerable effort and resources, but can provide highly accurate information for a specific location [25]. Of those studies, 11 had CPUE information that could be meaningfully compared with MRIP values generated by this study (S4 Table), i.e., gears were similarly described and it was possible to separate out CPUE for the same set of reef fishes (S5 Table). While there was a high degree of variation in CPUE among creel study locations, application of island-scale averages of CPUE values from those locations to the effort values estimated from this study would increase statewide catch estimates by ~50% [39]. The highest discrepancies between MRIP and creel CPUE values were for net fishing, which encompasses a diversity of net gears that were pooled into the net category. It would be desirable for MRIP to gather and maintain more detail on the gears used–particularly for net surveys–but more sophisticated groupings were not feasible for this analysis. It is difficult to know whether the generally higher CPUE values in creel surveys are indicative of MRIP underestimating CPUE, or whether the location of creel surveys might not be representative of the wide range of habitats and fisheries in Hawaiʻi, in particular, that creel surveys may have been more likely to occur at locations with locally important and established fisheries.

In a recent evaluation of the MRIP program, it was noted that a pilot mail survey generated higher fishing effort estimates than those derived from the MRIP telephone surveys and that an apparent decreasing trend in fishing participation may be an artifact of reliance on landline telephone surveys, that may not represent the population as a whole [37]. Underestimated effort by landline telephone surveys would lead to underestimation of reef fish catches. There is also the potential for nonresponse bias–that is, fishers are more likely to respond to the survey than non-fishers–which has been shown to overestimate fishing effort by 17%, in other states [40]. Hawaiʻi is atypical among U.S. coastal states, with its strong cultural importance placed on fishing and a large non-market subsistence fisheries sector [1,3,4]. Therefore it is encouraging that the MRIP survey approach in Hawaiʻi is currently being revised and may be substantially changed in the near future [36–38].

In Hawaiʻi, commercial fishers are required to report all of their catch, and therefore the commercial values reported here and elsewhere have no statistical error (i.e. they are not based on sampling). However, there is scope for inaccuracy, specifically under-estimation if licensed commercial fishers do not report all of their catch, or if unlicensed fishers sell part of their catch. In addition, a recent study of fish flow within the main Hawaiian Islands estimated unlicensed catch sold to distributers to be between 30 and 93,000 kg, i.e., 6–19% of the reported commercial catch [4]. Therefore, the reported commercial catch of nearshore reef-associated fisheries presented here is a conservative estimate.

Hawaiʻi is highly reliant on seafood for subsistence, cultural perpetuation, and food security [4]. In order to sustain fishing for subsistence, culture, recreation, and livelihoods, it is clearly important that non-commercial fishing activity is properly estimated. Improved fisheries data, available at more relevant spatial scales, would strengthen the scope for managers and communities to ensure that Hawaiʻi’s residents can achieve the full benefits of a long-term sustainable reef fishery.

## Supporting information

S1 Table Species classified as ‘reef fish’. (PDF)

S2 Table Gear types recorded in MRIP surveys. (PDF)

S3 Table Fishing effort: Average hours fished by gear per trip. (PDF)

S4 Table Main Hawaiian Islands creel survey information. (PDF)

S5 Table CPUE values for line, net, and spear. (PDF)

## References

[1] Pooley SG. Hawaii’s Marine Fisheries: Some History, Long-term Trends, and Recent Developments. Mar Fish Rev. 1993;55: 12.

[2] Schug DM. Hawaii’s commercial fishing industry: 1820–1945. Hawaii J Hist. 2001;35: 15–34.

[3] Friedlander AM, Shackeroff JM, Kittinger JN, Friedlander AM. Customary Marine Resource Knowledge and Use in Contemporary Hawai‘i. Pacific Sci. 2013;67: 441–460. doi: 10.2984/67.3.10

[4] Grafeld S, Oleson KLL, Teneva L, Kittinger JN. Follow that fish: Uncovering the hidden blue economy in coral reef fisheries. PLoS One. 2017;12: 1–25. doi: 10.1371/journal.pone.0182104 28771508 10.1371/journal.pone.0182104 PMC5542444

[5] Pauly D, Christensen V, Guénette S, Pitcher TJ, Sumaila UR, Walters CJ, et al Towards sustainability in world fisheries. Nature. 2002;418: 689–695. doi: 10.1038/nature01017 12167876 10.1038/nature01017

[6] Zeller D, Darcy M, Booth S, Lowe MK, Martell S. What about recreational catch? Fish Res. 2008;91: 88–97. doi: 10.1016/j.fishres.2007.11.010

[7] Pauly D, Zeller D. Accurate catches and the sustainability of coral reef fisheries. Curr Opin Environ Sustain. Elsevier B.V.; 2014;7: 44–51. doi: 10.1016/j.cosust.2013.11.027

[8] Friedlander AM, Nowlis J, Koike H. Improving Fisheries Assessments Using Historical Data: Stock Status and Catch LImits. Marine Hsitorical Ecology in Conservation. 2014.

[9] Nadon MO. Stock assessment of the coral reef fishes of Hawaii, 2016 2017.

[10] Nadon MO, Ault JS, Williams ID, Smith SG, Dinardo GT. Length-based assessment of coral reef fish populations in the main and Northwestern Hawaiian Islands. PLoS One. 2015;10: 1–19. doi: 10.1371/journal.pone.0133960 26267473 10.1371/journal.pone.0133960 PMC4534412

[11] Williams ID, Baum JK, Heenan A, Hanson KM, Nadon MO, Brainard RE. Human, oceanographic and habitat drivers of central and western pacific coral reef fish assemblages. PLoS One. 2015;10 doi: 10.1371/journal.pone.0120516 25831196 10.1371/journal.pone.0120516 PMC4382026

[12] Williams ID, Walsh WJ, Schroeder RE, Friedlander AM, Richards BL, Stamoulis KA. Assessing the importance of fishing impacts on Hawaiian coral reef fish assemblages along regional-scale human population gradients. Environ Conserv. 2008;35: 261 doi: 10.1017/S0376892908004876

[13] Juvik S, Juvik J. Atlas of Hawaii. 3rd ed Honolulu: University of Hawaii Press; 1998.

[14] Williams ID, Ma H. Estimating catch weight of reef fish species using estimation and intercept data from the Hawaii marine recreational fishing survey. 2013.

[15] Ma H, Ogawa T. Hawaii Marine Recreatinal Fishing Survey: A Summary of Current Sampling, Estimation, and Data Analyses. Honolulu; 2016. doi: 10.7289/V5/TM-PIFSC-55

[16] Wolter KM. Introduction to variance estimation Springer; 2007.

[17] Turner R, Cakacaka A, Graham N, Polunin N, Pratchett M, Stead S, et al Declining reliance on marine resources in remote South Pacific societies: ecological versus socio-economic drivers. Coral Reefs. 2007;26: 997–1008.

[18] Severance C, Franco R, Hamnett M, Anderson C, Aitaoto F. Effort Triggers, Fish Flow, and Customary Exchange in American Samoa and the Northern Marianas: Critical Human Dimensions of Western Pacific Fisheries. Pacific Sci. 2013;67: 383–393.

[19] Vaughan MB, Vitousek PM. Mahele: Sustaining Communities through Small-Scale Inshore Fishery Catch and Sharing Networks. Pacific Sci. 2013;67: 329–344. doi: 10.2984/67.3.3

[20] Laurans Y, Pascal N, Binet T, Brander L. Economic valuation of ecosystem services from coral reefs in the South Pacific: Taking stock of recent experience. J Environ Manage. 2013; Available: http://www.sciencedirect.com/science/article/pii/S0301479712006160 10.1016/j.jenvman.2012.11.031 23295680

[21] Bellwood DR, Hoey a. S, Hughes TP. Human activity selectively impacts the ecosystem roles of parrotfishes on coral reefs. Proc R Soc B Biol Sci. 2011;279: 1621–1629. doi: 10.1098/rspb.2011.1906 22090383 10.1098/rspb.2011.1906 PMC3282342

[22] Brander LM, Van Beukering P, Cesar HSJ. The recreational value of coral reefs: A meta-analysis. Ecol Econ. 2007;63: 209–218. doi: 10.1016/j.ecolecon.2006.11.002

[23] Kittinger JN, Teneva LT, Koike H, Stamoulis KA, Kittinger DS, Oleson KLL, et al From reef to table: Social and ecological factors affecting coral reef fisheries, artisanal seafood supply chains, and seafood security. PLoS One. 2015;10: 1–24. doi: 10.1371/journal.pone.0123856 26244910 10.1371/journal.pone.0123856 PMC4526684

[24] Everson A, Friedlander AM. Catch, Effort, and Yields for Coral Reef Fisheries in Kaneohe Bay, Oahu and Hanalei Bay, Kauai: Comparison between a large urban and small rural embayment. Status of Hawaii’s Coastal Fisheries in the New Millenium: Proceedings of the 2001 Fisheries Symposium American Fisheries Society, Hawaii Chapter. 2001. pp. 108–128.

[25] Delaney DG, Teneva LT, Stamoulis KA. Patterns in artisanal coral reef fisheries reveal best practices for monitoring and management. 2017; 10.7717/peerj.4089 PMC5719965 29226033

[26] Jupiter SD, Egli DP. Ecosystem-Based Management in Fiji: Successes and Challenges after Five Years of Implementation. J Mar Biol. 2011;2011: 1–14. doi: 10.1155/2011/940765

[27] Johannes RE. the Renaissance of Community-Based Marine Resource Management in Oceania. Annu Rev Ecol Syst. 2002;33: 317–340. doi: 10.1146/annurev.ecolsys.33.010802.150524

[28] Cinner JE, McClanahan TR, MacNeil M a., Graham N a. J, Daw TM, Mukminin a., et al Comanagement of coral reef social-ecological systems. Proc Natl Acad Sci. 2012;109: 5219–5222. doi: 10.1073/pnas.1121215109 22431631 10.1073/pnas.1121215109 PMC3325732

[29] Friedlander AM, Stamoulis KA, Kittinger JN, Drazen JC, Tissot BN. Understanding the Scale of Marine Protection in Hawai’i: From Community-Based Management to the Remote Northwestern Hawaiian Islands. Advances in Marine Biology. 2014 doi: 10.1016/B978-0-12-800214-8.00005–0 10.1016/B978-0-12-800214-8.00005-0 25358300

[30] Friedlander AM. A perspective on the management of coral reef fisheries In: Mora C, editor. Ecology of Fishes on Coral Reefs. Cambridge, UK: Cambridge University Press; 2015 pp. 208–214.

[31] Ayers AL, Kittinger J, Imperial M, Vaughan M. Making the transition to co-management governance arrange-ments in Hawai’i: A framework for understanding transaction and transformation costs. Int Joural Commons. 2017;11 doi: 10.18352/ijc.709

[32] Schemmel E, Friedlander AM, Andrade P, Keakealani K, Castro LM, Wiggins C, et al The codevelopment of coastal fisheries monitoring methods to support local management. Ecol Soc. 2016;21: art34 doi: 10.5751/ES-08818-210434

[33] Smith MK. An Ecological Perspective on Inshore Fisheries in the Main Hawaiian Islands. Mar Fish Rev. 1993;55: 34–49.

[34] Weijerman M, Williams I, Gutierrez J, Grafeld S, Tibbatts B, Davis G. Trends in biomass of coral reef fishes, derived from shore-based creel surveys in Guam. Fish Bull. 2016;114: 237–256. doi: 10.7755/FB.114.2.9

[35] Dalzell P. Catch rates, selectivity and yields of reef fisheries In: Polunin N, Roberts C, editors. Reef Fisheries. London: Chapman & Hall; 1996 p. 161:192.

[36] Hongguang Ma; Tom Ogawa; Demello J. A Review of the Current Sampling and Estimation Methods of the Hawaii Marine Recreational Fishing Survey (HMRFS). 2013; 1–28.

[37] Ma H, Ogawa TK, Sminkey TR, Breidt FJ, Lesser VM, Opsomer JD, et al Pilot surveys to improve monitoring of marine recreational fisheries in Hawaii. Fish Res. Elsevier; 2018;204: 197–208. doi: 10.1016/j.fishres.2018.02.010

[38] Ma H, Hamm D. Results of a Pilot Study to Improve Intercept Surveys in the Hawaii Marine Recreational Fishery. 2015.

[39] McCoy KS. Estimating nearshore fisheries catch for the main Hawaiian Islands University of Hawaii at Manoa 2015.

[40] Andrews R, Brick JM, Mathiowetz NA. Development and Testing of Recreational Fishing Effort Surveys: Testing a Mail Survey Design. 2014.

