# Supplementary material for: Estimating nearshore coral reef-associated fisheries production from the main Hawaiian Islands
Source: PLoS One. 2018 Apr 16;13(4):e0195840. doi: 10.1371/journal.pone.0195840 (PMC5901996; doi:10.1371/journal.pone.0195840)
Supplement: S1 Table — (PDF) [file pone.0195840.s001.pdf]

S1 Table. Species classified as 'reef fish'.

ACANTHURIDAE: ***Acanthurus achilles, Acanthurus blochii, Acanthurus dussumieri, Acanthurus nigroris, Acanthurus triostegus, Ctenochaetus hawaiiensis, Ctenochaetus strigosus, Naso hexacanthus, Naso lituratus,*** *Acanthurus leucopareius, Acanthurus nigrofuscus, Acanthurus olivaceous, Acanthurus xanthopterus, Naso annulatus, Naso brevirostris, Naso unicornis, Zebrasoma flavescens*
BALISTIDAE: *Melichthys niger, Melichthys vidua, Rhinecanthus aculeatus, Rhinecanthus rectangulus, Sufflamen bursa*
CARANGIDAE: ***Alectis ciliaris,*** *Carangoides ferdau, Caranx lugubris,* ***Carangoides orthogrammus, Caranx ignobilis, Caranx melampygus, Caranx sexfasciatus, Elagatis bipinnulata, Gnathanodon speciosus, Pseudocaranx cheilio, Scomberoides lysan, Seriola dumerili, Uraspis helvola***
CARCHARHINIDAE: *Carcharhinus amblyrhynchos, Carcharhinus galapagensis, Carcharhinus melanopterus, Carcharhinus plumbeus, Triaenodon obesus*
CHAETODONTIDAE: *Chaetodon lunula, Chaetodon unimaculatus*
CIRRHITIDAE: ***Cirrhitus pinnulatus,*** *Paracirrhites forsteri*
HOLOCENTRIDAE: ***Sargocentron spiniferum, Sargocentron tiere,*** *Myripristis amaena, Myripristis berndti, Myripristis chryseres, Myripristis vittata, Plectrypops lima, Sargocentron xantherythrum*
KYPHOSIDAE: *Kyphosus bigibbus, Kyphosus cinerascens, Kyphosus vaigiensis*
LABRIDAE: ***Oxycheilinus unifasciatus,*** *Anampses chrysocephalus, Anampses cuvier, Bodianus bilunulatus, Cheilio inermis, Coris flavovittata, Coris gaimard, Gomphosus varius, Halichoeres ornatissimus, Iniistius baldwini, Iniistius pavo, Iniistius umbrilatus, Novaculichthys taeniourus, Thalassoma ballieui, Thalassoma duperrey, Thalassoma trilobatum*
LETHRINIDAE: ***Monotaxis grandoculis***
LUTJANIDAE: ***Aphareus furca, Lutjanus fulvus, Lutjanus kasmira,*** *Aprion virescens*
MUGILIDAE: ***Mugil cephalus, Neomyxus leuciscus,*** *Valamugil engeli*
MULLIDAE: ***Mulloidichthys flavolineatus, Mulloidichthys vanicolensis, Parupeneus cyclostomus, Parupeneus insularis, Parupeneus multifasciatus, Parupeneus pleurostigma, Parupeneus porphyreus, Upeneus arge,*** *Mulloidichthys pfluegeri*
POLYNEMIDAE: ***Polydactylus sexfilis***
POMACENTRIDAE: ***Abudefduf sordidus,*** *Abudefduf abdominalis, Chromis verater, Stegastes fasciolatus,*
PRIACANTHIDAE: *Heteropriacanthus cruentatus, Priacanthus meeki*
SCARIDAE: ***Calotomus carolinus,*** *Chlorurus perspicillatus, Chlorurus sordidus, Scarus dubius, Scarus psittacus, Scarus rubroviolaceus*
SCORPAENIDAE: *Dendrochirus barberi, Scorpaenopsis cacopsis*
SERRANIDAE: *Cephalopholis argus*
SPHYRAENIDAE: ***Sphyraena barracuda, Sphyraena helleri***
EELS: *Conger cinereus, Enchelycore pardalis, Gymnomuraena zebra, Gymnothorax eurostus, Gymnothorax flavimarginatus, Gymnothorax rueppelliae, Uropterygius macrocephalus*
OTHERS: ***Albula glossodonta,*** *Apogon kallopterus,* ***Aulostomus chinensis,*** *Bothus mancus,* ***Chanos chanos,*** *Diodon holocanthus, Diodon hystrix, Fistularia commersonii, Kuhlia sandwicensis, Aluterus scriptus*

The above taxa were reported as having been caught in the MRIP intercept data between 2004-13, but species in bold were also present in commercial catch data reports. Note that a large portion of the commercial catch is recorded as miscellaneous or in higher taxonomic categories.
