# Supplementary material for: Estimating nearshore coral reef-associated fisheries production from the main Hawaiian Islands
Source: PLoS One. 2018 Apr 16;13(4):e0195840. doi: 10.1371/journal.pone.0195840 (PMC5901996; doi:10.1371/journal.pone.0195840)
Supplement: S2 Table — (PDF) [file pone.0195840.s002.pdf]

Table S2. Gear types recorded in MRIP surveys for catch/intercept surveys, and for effort/telephone surveys, and how gear was grouped to be comparable.

Combined gear	Catch/intercept	Effort/telephone	
boat and shore	boat and shore	boat gear	shore gear
line	handline, handpole, rod and reel	handline, rod and reel, casting, whipping	handline, kite, line, rod and reel, casting, spinning, dunking, whipping
net	scoop net, throw net, gill net, cross net, surround net	netting	netting
spear	spear	spear	spear
**Excluded gear (i.e. considered 'not-reef' gears)**			
glean	trawl	bottomfishing	gleaning
crab net	crab net	trolling	crabbing
deep	hukilau	jigging	trap

Gear types used in telephone and intercept surveys were not classified in the same ways; therefore, gear was consolidated into 3 categories: net, line, and spear. For telephone data, gear types of rod and reel, dunking, spinning, whipping, handline, casting, kite, and line were combined into the line category.
