# Supplementary material for: Estimating nearshore coral reef-associated fisheries production from the main Hawaiian Islands
Source: PLoS One. 2018 Apr 16;13(4):e0195840. doi: 10.1371/journal.pone.0195840 (PMC5901996; doi:10.1371/journal.pone.0195840)
Supplement: S3 Table — (PDF) [file pone.0195840.s003.pdf]

Table S3. Fishing effort: Average hours fished by gear per trip.

Island	Platform	Gear	Average hours	N	SD
Hawaii	Boat	Line	6.09	1395	3.08
Kauai	Boat	Line	6.73	2009	3.51
Maui	Boat	Line	5.72	2255	3.00
Molokai	Boat	Line	5.64	327	6.76
Oahu	Boat	Line	5.57	3624	2.55
Hawaii	Shore	Line	3.58	2012	4.01
Kauai	Shore	Line	2.86	2173	1.71
Maui	Shore	Line	3.95	2735	4.20
Molokai	Shore	Line	3.35	736	5.91
Oahu	Shore	Line	3.61	4679	2.33
Hawaii	Boat	Net	5.43	3	3.51
Kauai	Boat	Net	6.75	8	4.22
Maui	Boat	Net	5.43	1	NA
Molokai	Boat	Net	6.00	22	5.78
Oahu	Boat	Net	3.55	10	1.77
Hawaii	Shore	Net	2.23	171	3.18
Kauai	Shore	Net	1.68	28	1.06
Maui	Shore	Net	2.14	73	1.12
Molokai	Shore	Net	2.54	84	2.16
Oahu	Shore	Net	1.91	58	1.05
Hawaii	Boat	Spear	3.93	74	1.93
Kauai	Boat	Spear	4.11	19	1.17
Maui	Boat	Spear	3.68	88	2.80
Molokai	Boat	Spear	5.94	133	3.68
Oahu	Boat	Spear	3.70	383	1.88
Hawaii	Shore	Spear	2.78	173	4.55
Kauai	Shore	Spear	2.38	93	1.22
Maui	Shore	Spear	2.05	234	1.08
Molokai	Shore	Spear	2.59	119	1.37
Oahu	Shore	Spear	2.38	130	0.83
Lanai	Boat	Line	5.95	2255	3.00
Lanai	Shore	Line	3.47	2735	4.20
Lanai	Boat	Net	5.43	1	NA
Lanai	Shore	Net	2.10	73	1.12
Lanai	Boat	Spear	4.27	88	2.80
Lanai	Shore	Spear	2.43	234	1.08

Gear hours (line, net, or spear) by platform (boat or shore) for individual islands. Numbers are a yearly average from a 10-year data set. N = sample size, SD = standard deviation.
