# Supplementary material for: Estimating nearshore coral reef-associated fisheries production from the main Hawaiian Islands
Source: PLoS One. 2018 Apr 16;13(4):e0195840. doi: 10.1371/journal.pone.0195840 (PMC5901996; doi:10.1371/journal.pone.0195840)
Supplement: S4 Table — (PDF) [file pone.0195840.s004.pdf]

S4 Table. Main Hawaiian Islands creel survey information.

Location	Island	Month/Year	Duration	Coastline (km)	Organization	Source
Kahekili	Maui	1/2011-12/2011	1 year	~ 3	HCFRU, UH; HDAR	Friedlander AM, Koike H, Kekoa L, Sparks R. Design, development, and implementation of a survey of the fisheries of the Kahekili Herbivore Fisheries Management Area. 2011.
Wailuku	Maui	3/2013 - 8/2013	5 months	~3.5	FERL UH; Wailuku Community Managaed Makai Area	Koike H, Carpio J, Friedlander AM. Final Creel Survey Report for Wailuku Community Management Area, Maui County, Hawaii. 2014.
Haena	Kauai	12/2009-12/2010	1 year	3.8	Makai Watch Haʻena; FERL, UH	Raw data
Hanalei	Kauai	7/1992-12/1993	~1 year	~ 4.8	HCFRU, UH; HDAR	Everson A, Friedlander AM. Catch, Effort, and Yields for Coral Reef Fisheries in Kaneohe Bay, Oahu and Hanalei Bay, Kauai: Comparison between a large urban and small rural embayment. Status of Hawaii's Coastal Fisheries in the New Millennium: Proceedings of the 2001 Fisheries Symposium American Fisheries Society, Hawaii Chapter. 2001. pp. 108–128.
Kiholo	Hawaii	4/2012-5/2013	1 year	~4.7	HCFRU, UH; Alakaʻi Consulting LLC; Hui Aloha Kiholo	Kittinger JN, Teneva LT, Koike H, Stamoulis KA, Kittinger DS, Oleson KLL, et al. From reef to table: Social and ecological factors affecting coral reef fisheries, artisanal seafood supply chains, and seafood security. PLoS One. 2015;10: 1–24. doi:10.1371/journal.pone.0123856
Kaupulehu	Hawaii	8/2013-11/2013	3 months	3.5	FERL, UH; TNC	Koike H, Wiggins C, Most R, Conklin E, Minton D, Friedlander AM. Final Creel Survey Report for Kaʻūpūlehu Creel Survey Project, North Kona, Hawaiʻi Island. 2015.
Puako	Hawaii	12/2008-12/2009	1 year	3.6	UH, TNC	Raw data

Maunalua	Oahu	12/2007-11/2008	1 year	11	TNC; FERL, UH	Raw data
Waikiki	Oahu	6/1998-8/2001	2 years, 2 mo.	7	UH	Meyer CG. An empirical evaluation of the design and function of a small marine reserve. University of Hawaii at Manoa. 2003.
Kaneohe Bay	Oahu	1991 - 1992	1 year	12.8	NOAA PIFSC; NOAA NOS; OI	Everson A, Friedlander AM. Catch, Effort, and Yields for Coral Reef Fisheries in Kaneohe Bay, Oahu and Hanalei Bay, Kauai: Comparison between a large urban and small rural embayment. Status of Hawaii's Coastal Fisheries in the New Millennium: Proceedings of the 2001 Fisheries Symposium American Fisheries Society, Hawaii Chapter. 2001. pp. 108–128.
Kaneohe Marine Corps Base	Oahu	Feb-11	1 month	7 sites across 12.5 km	Pono Pacific Land Management LLC; NOAA PIFSC	Carnevale M, Allen S. Preliminary Assessment Concerning both the Shoreline Fishing Perceptions and the Quantitative Shoreline Fishing Effort, Harvest, and Catch at Marine Corps Base Hawaii; Kaneohe, Hawaii. 2011.

Survey site locations, time frame, duration, length of coastline surveyed, organization that conducted the surveys, and source. TNC (The Nature Conservancy), FERL (Fisheries Ecology Research Lab), UH (University of Hawaii), NOAA (National Oceanic and Atmospheric Administration), PIFSC (Pacific Islands Fisheries Science Center), NOS (National Ocean Service), OI (The Oceanic Institute), HDAR (Hawaii's Division of Aquatic Resources), HCFRU (Hawaii Cooperative Fishery Research Unit). The above information is a condensed version of information provided in [1], which collated and synthesized data from the 11 creel survey programs conducted in MHI, for which comparable data were available.

References

1. McCoy KS. Edtimating nearshore fisheries catch for the Main Hawaiian Islands. University of Hawaii at Manoa, MSc Thesis. 2015.
