# Supplementary material for: Estimating nearshore coral reef-associated fisheries production from the main Hawaiian Islands
Source: PLoS One. 2018 Apr 16;13(4):e0195840. doi: 10.1371/journal.pone.0195840 (PMC5901996; doi:10.1371/journal.pone.0195840)
Supplement: S5 Table — (PDF) [file pone.0195840.s005.pdf]

S5 Table. CPUE values for line, net, and spear.

Survey Period	Location	CPUE (kg/g hr)		
		Net	Line	Spear
2011	Kahekili, Maui	0.03	0.09	0.03
5/2013 - 5/2014	Wailuku, Maui	-	0.07	0.01
2010	Haena, Kauai	0.43	0.09	0.56
1993	Hanelei, Kauai	1.32	0.07	0.95
2009	Puako, Hawaii	0.84	0.14	0.36
8/2013-8/2014	Kaupulehu, Hawaii	0.39	0.33	0.37
5/2012-4/2013	Kiholo, Hawaii	1.81	0.89	1.79
1991-1992	Kaneohe Bay, Oahu	-	0.31	0.45
2008	Maunalua, Oahu	0.21	0.23	0.51
Feb-11	Kaneohe MCBH, Oahu	-	0.07	0.24
6/1998-8/2001	Waikiki, Oahu	-	0.04	1.13

Values come from creel surveys described in Table S4. Survey period is year the survey was conducted, location indicates the specific location for the creel surveys, and CPUE is listed for net, line, and spear. Due to the nature of these creel surveys, intercepts and therefore estimated CPUE values are for shore-based fishing [1].

References

1. McCoy KS. Edtimating nearshore fisheries catch for the Main Hawaiian Islands. University of Hawaii at Manoa, MSc Thesis. 2015.
